# QFMatch: multidimensional flow and mass cytometry samples alignment

**DOI:** 10.1038/s41598-018-21444-4

**Published:** 2018-02-19

**Authors:** Darya Y. Orlova, Stephen Meehan, David Parks, Wayne A. Moore, Connor Meehan, Qian Zhao, Eliver E. B. Ghosn, Leonore A. Herzenberg, Guenther Walther

**Affiliations:** 1 Department of Genetics, Stanford University School of Medicine, Stanford, CA USA; 2 Department of Mathematics, California Institute of Technology, Pasadena, CA USA; 3 Department of Statistics, Stanford University, Stanford, CA USA; 4 Department of Medicine, Emory University School of Medicine, Atlanta, GA USA

## Abstract

Part of the flow/mass cytometry data analysis process is aligning (matching) cell subsets between relevant samples. Current methods address this cluster-matching problem in ways that are either computationally expensive, affected by the curse of dimensionality, or fail when population patterns significantly vary between samples. Here, we introduce a quadratic form (QF)-based cluster matching algorithm (QFMatch) that is computationally efficient and accommodates cases where population locations differ significantly (or even disappear or appear) from sample to sample. We demonstrate the effectiveness of QFMatch by evaluating sample datasets from immunology studies. The algorithm is based on a novel multivariate extension of the quadratic form distance for the comparison of flow cytometry data sets. We show that this QF distance has attractive computational and statistical properties that make it well suited for analysis tasks that involve the comparison of flow/mass cytometry samples.

## Introduction

Most flow and mass cytometry applications in biomedical studies are based on comparisons between/among control and test samples. Dissimilarities between/among samples may be due to drug treatment regime, progression of disease, response to therapies, etc. To define these dissimilarities across samples, the populations of cells in each sample are usually clustered to reveal phenotypically distinct cell subsets that can then be matched, quantified and compared between samples.

Traditionally, this type of cluster analysis has been done by manually gating the data into arbitrary clusters. These methods have proven effective in a gross sense but are both subjective and extremely laborious, making them difficult to apply with current high-dimensional (Hi-D) data sets. The need to facilitate these analyses, and make them more accurate, has motivated development of automated clustering and cluster matching methods for Hi-D flow and mass cytometry data.

Both of these tasks (cluster identification and cluster matching) are highly challenging because they are subject to the “curse of dimensionality”, a well-known statistical problem for Hi-D data that compromises both statistical validity and computational performance^1,2^. Here, we discuss the limitations of currently available methods for cluster matching applications, and demonstrate that employing a multivariate extension of the quadratic form distance^3^ overcomes key limitations.

Existing methods address the cluster matching problem in two different ways, both of which have limitations. The first way is clustering one sample at a time and aligning/matching the cell subsets (clusters) present in multiple samples postclustering (e.g., as is done in the FLAME analysis^4^ and flowMatch package^5^). This conventional approach allows fast computational implementations in low dimensions. However, this approach can fail if the locations of the populations (clusters) significantly vary from sample to sample, or if populations disappear or appear between samples. Further, when clustering is performed in Hi-D settings, this approach can be compromised by the curse of dimensionality.

The second approach (e.g., Joint Clustering and Matching^6^, ASPIRE^7^) alleviates some of these problems by creating a Hi-D template of meta-clusters (distinct biologically-relevant cell types) in which all sample data are pooled, simultaneously clustered and then matched.

With these methods, multiple samples are treated as different realizations of a single underlying model reflecting the biological reality. However, apart from being computationally expensive, the majority of methods in this category identify clusters by fitting mathematical models to datasets. The feasibility of fitting in these cases, however, is dramatically affected by the curse of dimensionality, since the number of combinations of possible parameters to be considered increases dramatically as the number of dimensions increases above three or four.

Thus, although the existing methods offer solutions to some aspects of the cluster-matching problem, they still do not fully accommodate real-world flow/mass cytometry data. To pave the way toward a more robust solution of this problem, we developed QFMatch - a cluster matching method based on the quadratic form (QF) distance measure. QFMatch matches cell subsets (clusters) present in multiple samples postclustering. However, it accommodates cases when the location of a population varies significantly from sample to sample in two-dimensional display, or when populations disappear or appear between samples.

The key to our methodology is a new multivariate version of the quadratic form distance for the comparison of flow cytometry samples. Since such comparisons are a fundamental part of the analysis these data, there has been an active interest in developing suitable distance measures^3,4,8–10^. The quadratic form distance has several properties that make it an attractive candidate for these tasks: it is easy to implement, it can be computed quickly, and, as will be shown below, it possesses certain properties that are relevant for a meaningful comparison of flow cytometry distributions.

## Results

### The QF Distance

As pointed out in Orlova *et al*.^10^, a dissimilarity measure needs to possess certain properties in order to provide a biologically meaningful comparison between flow cytometry samples. In particular, it needs to satisfy the properties of a metric as well as a continuity property: small changes in subset location (e.g., due to instrument drift) or subset frequency should be reflected as only small changes in the dissimilarity measure. This continuity property makes it possible to distinguish biologically relevant differences from small differences due to instrument drift and other irrelevant factors. Orlova *et al*.^10^ point out that this requirement rules out the use of  dissimilarity measures based on p-values from many standard statistical tests, and they demonstrate that the Earth Mover’s Distance (EMD) is a suitable dissimilarity measure for comparing biomarker expression levels in cell populations. Unfortunately, the EMD is computationally intensive. Bernas *et al*.^3^ propose to use the quadratic form (QF) distance (Hafner *et al*.^11^) to quantify the dissimilarity between two univariate histograms of flow cytometry data:

1 $${D}^{2}({\bf{h}},{\bf{f}})={({\bf{h}}-{\bf{f}})}^{T}{\boldsymbol{A}}({\bf{h}}-{\bf{f}})=\sum _{i=1}^{n}\sum _{j=1}^{n}{a}_{ij}({h}_{i}-{f}_{i})({h}_{j}-{f}_{j})$$

Here *h*_*i*_ and *f*_*i*_ are the relative frequencies of the two histograms pertaining to the histogram bin indexed by i. That is, the two histograms employ the same bins and

2 $$\,{\sum }_{i}{h}_{i}={\sum }_{i}{f}_{i}=1$$

The matrix ***A*** = [*a*_*ij*_] is chosen to reflect the spatial dissimilarity between bins *i* and *j*. Note that in order to be a metric, *D*^2^(**h**, **f**) needs to be nonnegative, which restricts the choices for the similarity matrix ***A***. (Positive definiteness of ***A*** is sufficient but not necessary as

3 $${\sum }_{i}({h}_{i}-{f}_{i})=0$$

see Hafner *et al*.^11^). We point out that not all of the choices of ***A*** proposed in Bernas *et al*.^3^ satisfy this condition. Here we show that an appropriate choice of ***A*** not only guarantees that the QF is nonnegative, but that it futhermore results in a monotonic behavior that mirrors the continuity condition given above. We also show how the QF distance can be extended in a computationally simple way to a multivariate, even high-dimensional, situation. Therefore this QF distance shares the advantagous properties of the EMD, but it is conceptually much simpler to implement and, importantly, it can be computed quickly.

In more detail, we propose to use

4 $${{\rm{a}}}_{{\rm{ij}}}=1-{{\rm{d}}}_{{{\rm{M}}}_{{\rm{ij}}}}/{{\rm{d}}}_{{\rm{\max }}}$$

where ${{\rm{d}}}_{{{\rm{M}}}_{{\rm{ij}}}}$ is the Euclidean distance between centers of mass (calculated on combined samples) of the *i*th and *j*th bins, and d_max_ is the maximum value of all the ${{\rm{d}}}_{{{\rm{M}}}_{{\rm{ij}}}}$. Note that this is a generic definition that applies to the univariate as well as the multivariate setting; we will discuss an appropriate binning scheme below. It then follows from a result in Hafner *et al*.^11^ that for this choice of ***A*** the QF distance *D*^2^(**h**,**f**) is always nonnegative. Furthermore, employing a matrix **A** with off-diagonal elements that depend on the spatial distance between the bins in a suitable way, such as a_ij_ given above, has the effect that *D*^2^(**h**,**f**) not only increases with the magnitude of |**h**-**f**|, but also with the spatial distance of the non-zero elements of **h**-**f**. While this is not exactly mathematically equivalent to the continuity condition stated above (one can mathematically construct counterexamples where the continuity condition fails, but these counterexamples appear not to be practically relevant), it results in the desired behavior of *D*^2^(**h**,**f**) that allows a biologically meaningful quantification of the difference between the two samples as is demonstrated with experimental results below. This property is not shared by some other common distance measures such as the chi-square distance

5 $${\sum }_{i}{({h}_{i}-{f}_{i})}^{2}/({h}_{i}+{f}_{i})$$

which may fail to increase even as the spatial distance between the two populations increases, see Tables 1 and 2 (corresponding code is available at https://github.com/zq00/QFMatch-simulation).

**Table 1:** Values of EMD, QF and chi-square distance between n = 10,000 data simulated from N(0,I) and n data simulated from (1 − p) N(0,I) + p N(u,I) (6) in dimensions 2 and 20, for various values of p and u.

dim = 2					dim = 20				
p	u	EMD	QF	Chi-square	p	u	EMD	QF	Chi-square
0	0	0.045 (0.004)	0.007 (0.002)	0.102 (0.005)	0	0	0.435 (0.010)	0.0118 (0.001)	0.1023 (0.005)
0.001	1	0.044	0.007	0.102	0.001	1	0.437	0.0117	0.1030
0.001	5	0.044	0.007	0.098	0.001	5	0.431	0.0109	0.0997
0.001	10	0.048	0.007	0.103	0.001	10	0.438	0.0120	0.1033
0.01	1	0.044	0.007	0.102	0.01	1	0.426	0.0119	0.0986
0.01	5	0.076	0.010	0.110	0.01	5	0.439	0.0128	0.1027
0.01	10	0.128	0.012	0.116	0.01	10	0.474	0.0125	0.1044
0.1	1	0.104	0.025	0.11	0.1	1	0.438	0.0170	0.10
0.1	5	0.503	0.083	0.198	0.1	5	0.783	0.0756	0.1848
0.1	10	0.985	0.104	0.20	0.1	10	1.243	0.1018	0.2005
1	1	0.995	0.249	0.488	1	1	1.153	0.1479	0.3779
1	5	4.996	0.802	1.961	1	5	5.431	0.7503	1.9466
1	10	9.993	1.031	2.000	1	10	10.247	1.0307	2.000

The values for p = 0 are the averages over 100 simulations with the standard deviation given in brackets. Using the “average plus one standard deviation’ rule as a threshold for deciding when two distributions are different, one sees that EMD and QF behave similarly: they require about the same threshold for u to detect that a difference is present, and they increase monotonically with u thereafter. In contrast, the chi-square statistic often needs a higher threshold and shows essentially no increase from u = 5 to u = 10.

**Table 2:** Same as Table 1 but with sample size n = 100,000.

dim = 2					dim = 20				
p	u	EMD	QF	Chi-square	p	u	EMD	QF	Chi-square
0	0	0.017	0.001	0.161	0	0	0.529	0.0032	0.165
0.001	1	0.015	0.001	0.165	0.001	1	0.525	0.0033	0.162
0.001	5	0.019	0.002	0.165	0.001	5	0.528	0.0031	0.165
0.001	10	0.026	0.002	0.16	0.001	10	0.528	0.0033	0.164
0.01	1	0.021	0.003	0.163	0.01	1	0.534	0.0034	0.168
0.01	5	0.055	0.008	0.173	0.01	5	0.532	0.0048	0.165
0.01	10	0.108	0.01	0.175	0.01	10	0.598	0.011	0.176
0.1	1	0.104	0.024	0.171	0.1	1	0.54	0.011	0.167
0.1	5	0.497	0.076	0.255	0.1	5	0.855	0.065	0.247
0.1	10	1.001	0.1	0.263	0.1	10	1.353	0.099	0.264
1	1	0.996	0.227	0.537	1	1	1.151	0.125	0.434
1	5	4.993	0.755	1.964	1	5	5.425	0.665	1.957
1	10	10	0.988	2	1	10	10.225	0.961	2

Only one simulation was performed in the null case p = 0 due to the computational burden of EMD. The results confirm the conclusions of Table 1.

The relative frequencies *h*_*i*_ and *f*_*i*_ for bin i are computed once the k-dimensional measurement space is partitioned into bins. We propose to use adaptive binning^8^ on the combined sample, i.e. we merge the two samples for the construction of the bins only. Adaptive binning is a method for dividing k-dimensional data into bins such that all bins contain the same number of events. This strategy results in bins of variable size that “adapt” to the structure of the data. The algorithm begins by calculating the median and variance of the data for each of the k-dimensions included in the comparison. Next, we select the dimension j with the maximum variance and divide the data in half along the median value of that parameter, such that each bin contains an equal number of data points. The algorithm proceeds recursively until a predefined threshold is met (e.g., minimum number of data points per bin). This results in a collection of k-dimensional hyper-rectangular bins, with each bin containing an equal number of data points. This recursive binning scheme is quite straightforward to implement and can be computed very fast, with the dimension k of the measurement space affecting the computational complexity only linearly.

### Experimental performance of the QF distance and comparison with EMD and the chi-square distance

**Figure 1:**
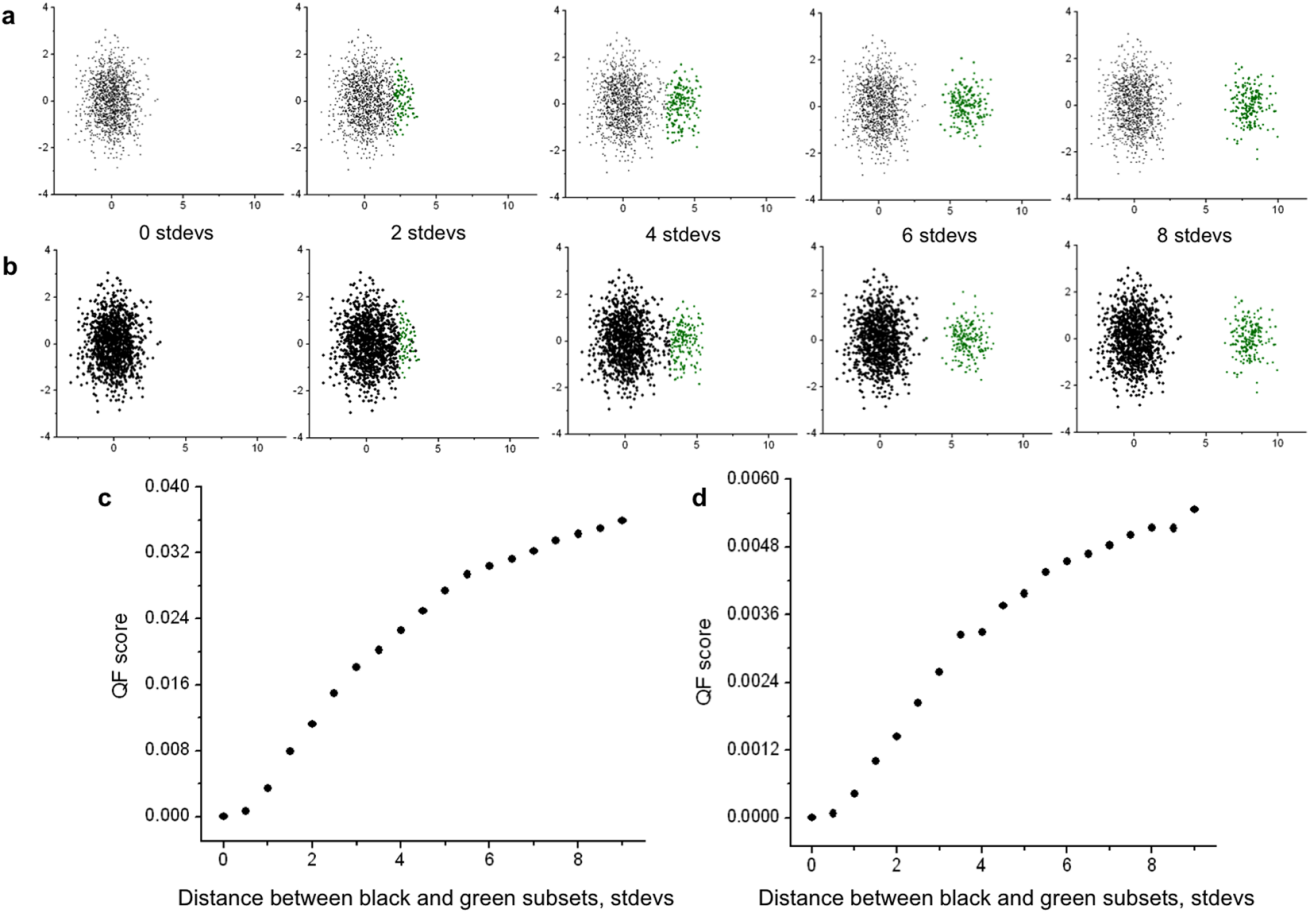
QF score increases smoothly and monotonically with the growing separation between two populations. Panels (a) and (b) of Fig. 1 show two normal distributions: a large population (black) and a smaller population (green). The green population (200 events) starts with a mean at the same position as the black population, and increases along the x axis in fixed increments (2 standard deviations) in each of the successive panels. The black population in panel a is three times smaller (1000 events) than the black population in panel b (3000 events). At each step, we calculate the QF dissimilarity score between the first panel “0 stdevs” and the joint distribution of the main (black) population with stimulated population (green). As the green population moves further from the black population, the QF score increases monotonically (panels c and d correspond to panels a and b, respectively).

Using synthetic datasets (Fig. 1a and b), we have verified that the QF score increases smoothly and monotonically with the growing separation between two subsets (see Fig. 1c and d). This property of the QF is very important, since it ensures that small differences between clusters (subsets) either in subset location (e.g., due to instrument drift) or subset frequency will be reflected as small changes in the QF score. In general, this is a critical property for cluster matching approaches designed to analyze flow cytometry and similar datasets, where small changes due to instrument noise, calibration, etc. are very common. This property insures that biologically similar samples with minor data aberrations, e.g., caused by shifts in flow instrument configuration during data collection, will still be well aligned.

We conducted a simulation study to compare the performance of QF with the EMD^10^ and the chi-square distance (equation 5) which is a popular dissimilarity score. We evaluated these three distance measures for comparing simulated data from a multivariate standard normal distribution N(0,I) with simulated data from

6 $$(1-{\rm{p}})\,{\rm{N}}(0,{\rm{I}})+{\rm{p}}\,{\rm{N}}({\rm{u}},{\rm{I}})$$

which represents the situation where a subset consisting of a fraction p of the data was moved by an amount u. Tables 1 and 2 give the values for these dissimilarity scores for various choices of p and u, and for sample sizes 10,000 and 100,000 in dimensions 2 and 20. It is seen that QF behaves quite similarly to EMD, both in its monotonic behavior as u increases as well as in the threshold that u needs to exceed before one can confidently declare that the two distributions are different. In contrast, the chi-square distance is less sensitive to detect this difference for small u, and it is also less able to discriminate large values of u. For example, the chi-square distance is very close to 2 for both u = 5 and u = 10. This is a drawback that is inherent in the definition of the chi-square distance: it will not reflect the size of the spatial separation if the two populations do not overlap. This makes the chi-square distance ill-suited for the comparison of flow and mass cytometry data.

The simulation study supports the conclusion that the QF shares the favorable properties of the EMD for the comparison of flow and mass cytometry data while having the advantage that it is conceptually much simpler to implement and that it can be computed much faster.

### The QF Match algorithm for multidimensional cluster alignment

**Figure 2:**
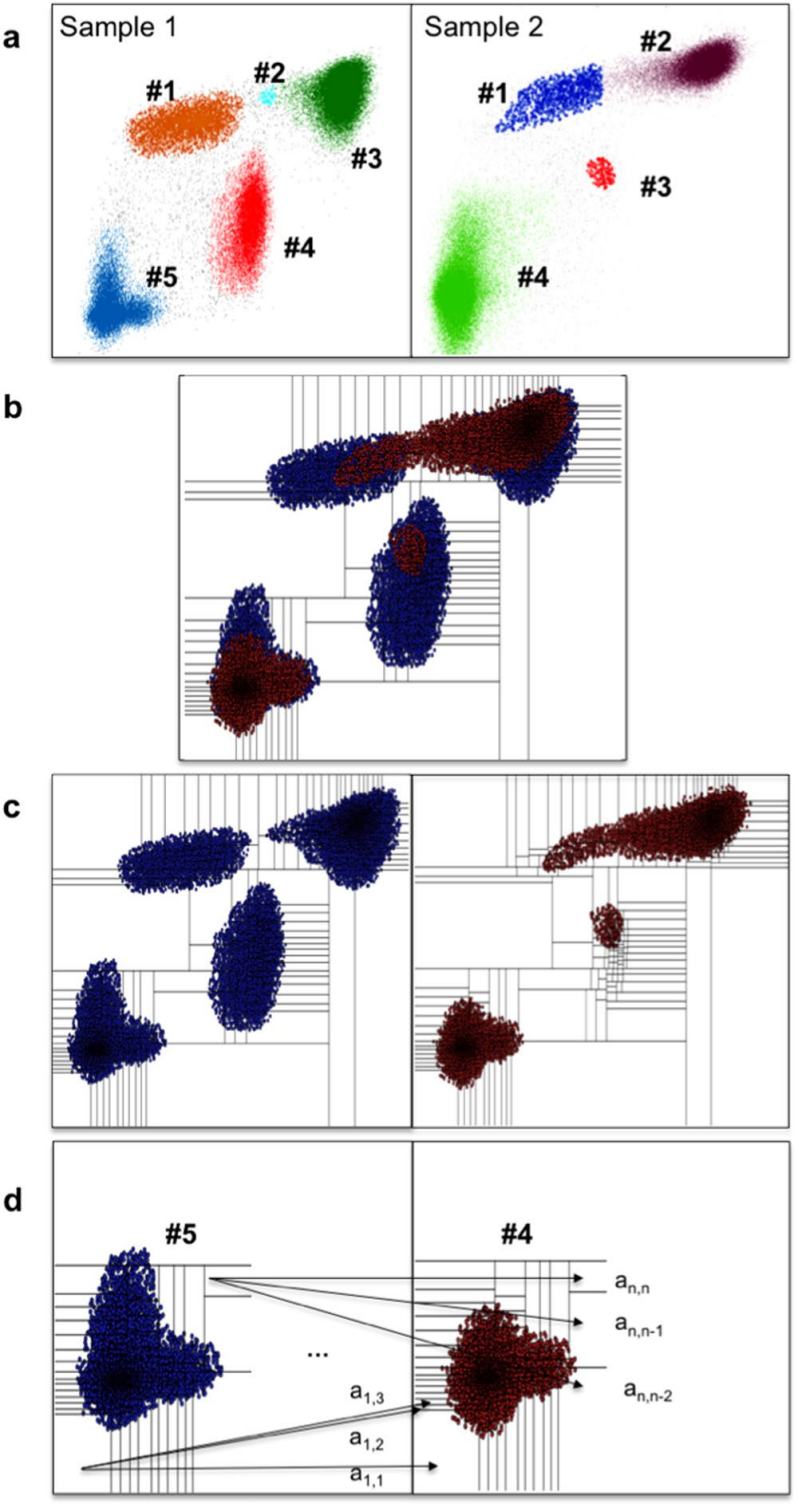
The steps of the QFMatch algorithm as applied in aligning one pair of clusters. Merge the beforehand clustered samples (panel a, colors distinguish subsets) and perform adaptive binning (panel b). Separate the merged binned samples into the original samples but preserve the binning pattern (panel c). Calculate QF dissimilarity between two clusters (panel d).

We now apply the multivariate QF distance to align subsets (clusters, subpopulations) between two samples. That is, the algorithm will take as input two flow cytometry samples, each of which has been subset beforehand either by a manual or an automated gating algorithm (Fig. 2a). Our QFMatch algorithm for cluster alignment consists of six steps:

*Step 1*: We do adaptive binning^8^ on the combined samples as decribed in the previous section (Fig. 2b).

*Step 2*: We apply the binning pattern derived in Step 1 to each of the two samples (Fig. 2c). For each cluster in each sample, we then construct a histogram using the bins from Step 1. (Thus each histogram pertaining to a cluster has total relative frequency equal to 1.)

**Figure 3:**
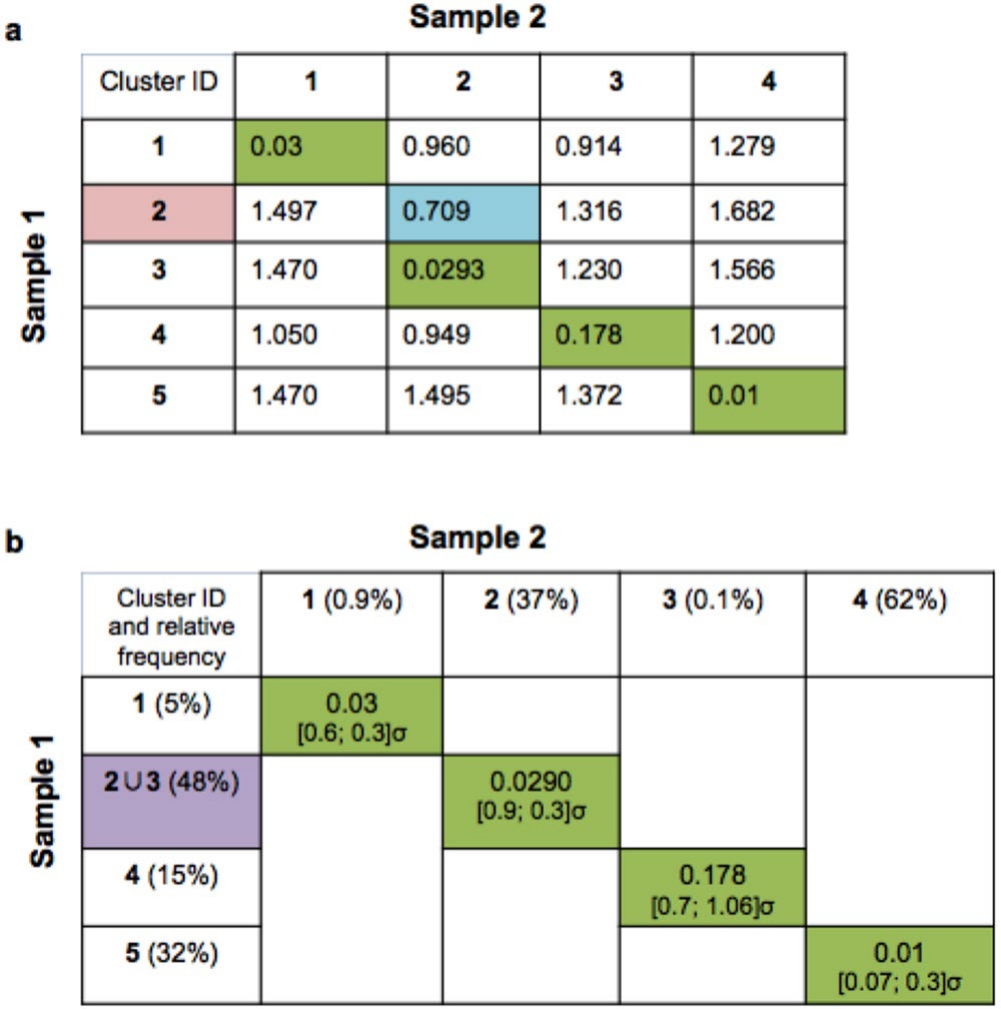
Pairwise QF-based dissimilarity scores. Panel a: we calculate the QF dissimilarity score for each possible combination of cluster pairs from Fig. 2a. Pairs with the smallest dissimilarity scores are marked in green and considered as matched. The cluster id of the merging candidate is marked in pink and its corresponding dissimilarity score is marked in blue. Panel b: if the initial dissimilarity score decreases as a result of the merging process, the presence of a cluster split is indicated (marked in violet); if not, then the unmatched cluster is considered as missing. The dissimilarity score for matched clusters are accompanied by the relative frequency of clusters and by the distance between geometric means of matched clusters in each dimension (expressed in standard deviation (σ) units of the cluster with which we match, i.e., the corresponding cluster from the Sample 1 in this example). For example, cluster #4 represents 62% of Sample 2 and its geometric mean is just 0.07 σ away in one dimension and 0.3 σ away in the other dimension from cluster #5 that represents 32% of Sample 1.

*Step 3*: For each combination of two clusters, where one cluster is from sample 1 and the other is from sample 2, we calculate a dissimilarity score based on the quadratic form distance, see Fig. 3.

*Step 4*: We treat the cluster pairs with the smallest dissimilarity score (marked in green on Fig. 3a) as matched. The remaining clusters in each sample are automatically treated as merging candidates (cluster id is marked in pink on Fig. 3a) for the clusters in the same sample. During this process, each merging candidate is combined with its nearest cluster in the same sample (i.e., both clusters have the smallest dissimilarity score to one of the clusters from the other sample, marked in blue on Fig. 3a). The dissimilarity score is then recalculated again.

*Step 5*: A decrease in the initial dissimilarity score as a result of the merging process on *Step 4* indicates that the cluster was split (marked in violet on Fig. 3b). The increase of dissimilarity score values that occurs as a result of the merging process indicate missing cluster(s).

*Step 6:* For each pair of matched clusters, we add information regarding relative frequency and the distance between geometric means of these clusters in each dimension (Fig. 3b). This helps to accommodate cases when for example each sample has only one subset and these subsets belong to different categories (i.e., different cell populations).

In our current implementation, each pair of matched clusters can be further compared to reveal global (dis)similarity by sequentially matching these clusters in each possible combination of the most informative dimension pairs (see Results section).

### The algorithm sensitivity and performance

**Figure 4:**
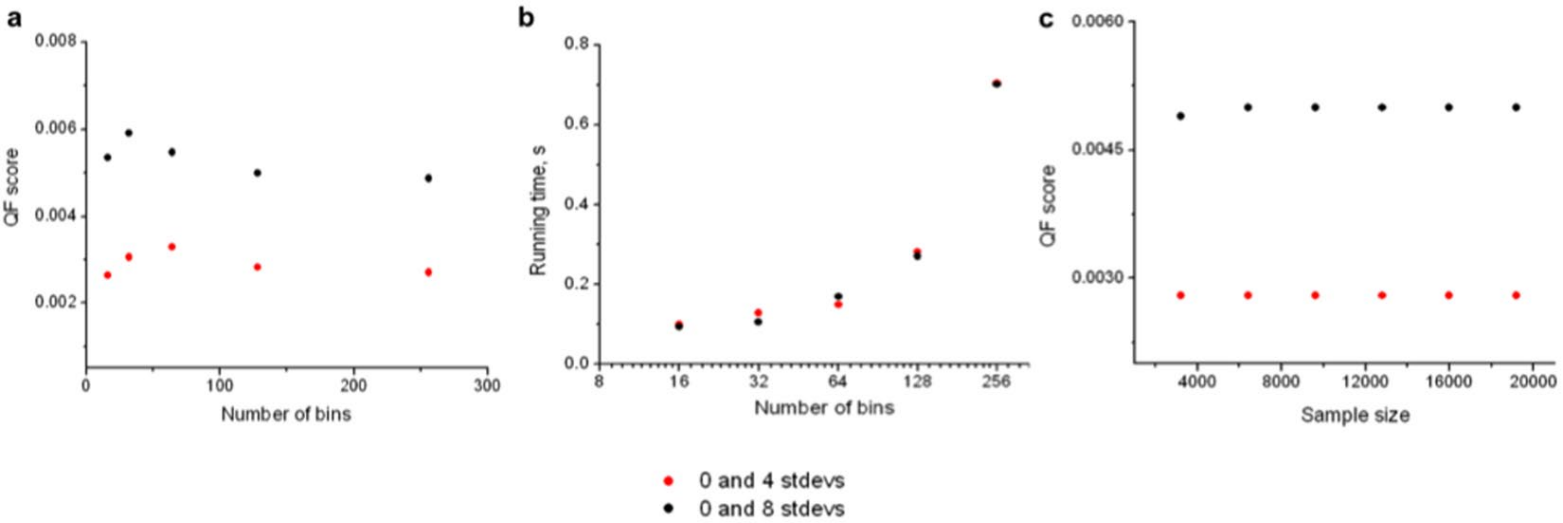
The sensitivity and performance of QFMatch algorithm. The effect of the number of bins on QF score (panel a) and on the running time (panel b, x-axis is in log_2_ scale). Variations in sample size do not affect the QF score (panel c).

To assess the sensitivity of QF to binning parameters, we randomly picked and analyzed three samples (“0 stdevs”, “4 stdevs”, and “8 stdevs”) from the synthetic dataset (Fig. 1b) for a range of bin sizes. The absolute event count for each sample is 3200 (which becomes 6400 when samples are merged for the binning step, see Fig. 2b). We binned the data using n = 16; 32; 64; 128; and 256 bins and plotted the results for QF score (Fig. 4a) and the corresponding running time (Fig. 4b). Running time increased linearly with the number of bins while QF values remained relatively constant.

Overall, these data indicate that QFMatch is robust in the choice of the number of bins. However, choosing the appropriate number of bins is a tradeoff between the algorithm’s speed and the binning resolution of the sample. Matching samples that contain small populations of cells may require finer binning than matching samples with larger populations only. For samples that contain small cell populations (e.g., cluster #3 in Sample 2, Fig. 2a), we chose a number of bins such that there are 2log_2_N events per bin, where N is the number of events in the smallest cell subset.

In this study, all running time calculations for the QFMatch algorithm implemented in Python (https://github.com/dyorlova/QFMatch; MATLAB implementation is available at http://cgworkspace.cytogenie.org/GetDown2/domains/FACS/QFMatchStandAlone.pdf) were performed on a 3.1 GHz Intel Core i7 with 16 GB of RAM running Mac OS X 10.11.6. With this implementation, it took a few seconds to cluster data with a two-dimensional density-based merging (DBM) algorithm^12^ and 14 seconds to align BALB/c with RAG^−/−^ (~268 000 cells total, see Results section below for more detail about these samples) with QFMatch using 256 bins. This example provides a sense of QF-based cluster matching algorithm speed in our current implementation.

We also showed that the QF dissimilarity score is invariable with sample size (Fig. 4c). Thus, we increased the size of “0 stdevs”, “4 stdevs”, and “8 stdevs” from the original sample size (3200 events including 200 events corresponding to the small green population, see Fig. 1b) 2–6 times and aligned samples “4 stdevs” and “8 stdevs” with “0 stdevs” sample using 128 bins.

### Appying the QFMatch cluster alignment algorithm utility to flow cytometry data

We used three real datasets to demonstrate how the analysis pipeline, which includes the QF-based cluster matching aglorithm, can be used to do automated clustering and alignment of cell populations identified in flow cytometry data. The same data analysis workflow was used in the three examples discussed below (see Materials and Methods).

### Matching of cell subsets between patient samples, even when relative cell frequencies differ by one order of magnitude and marker expression levels vary between patients

Here we present two examples demonstrating that QF-based algorithm successfully matches cell subsets that vary significantly between samples.

**Figure 5:**
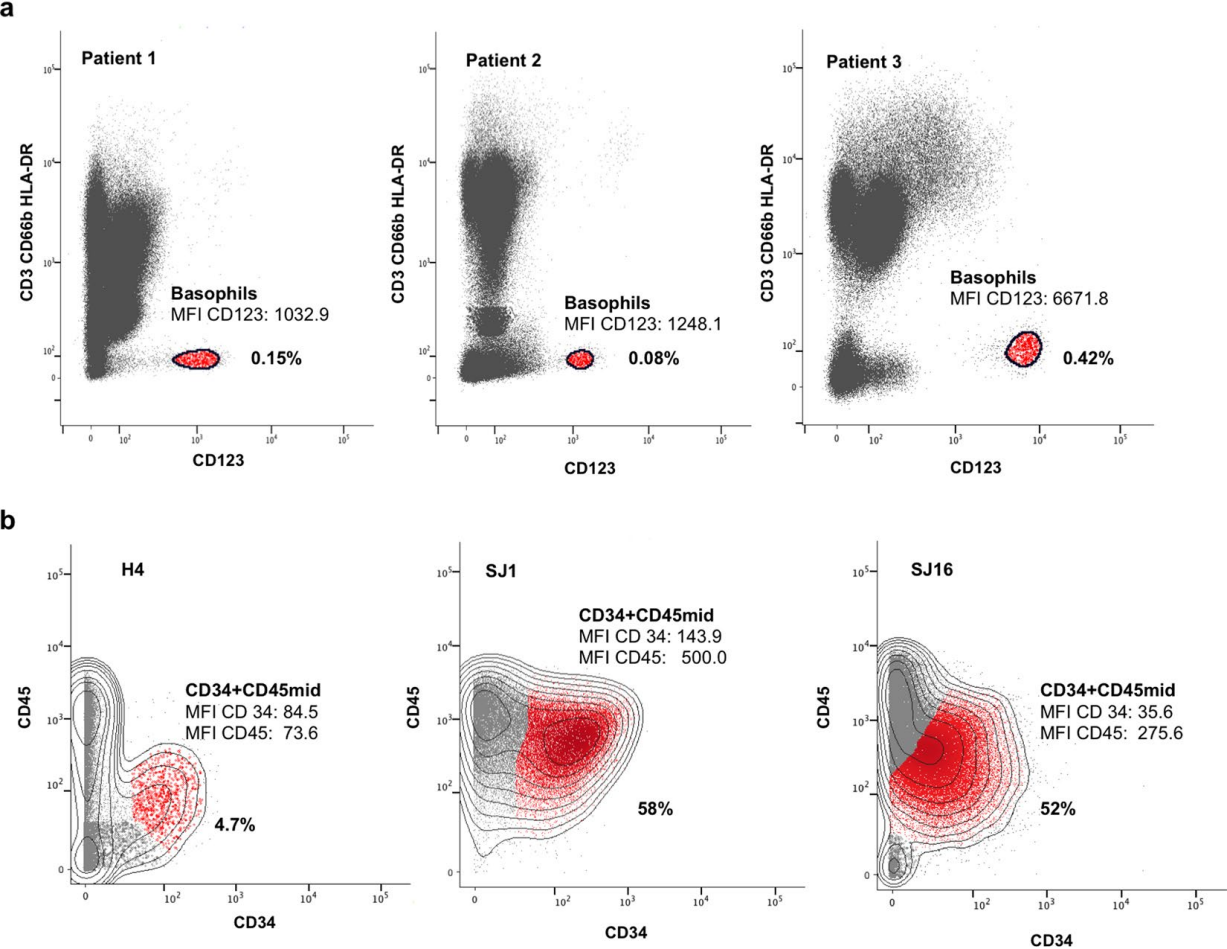
The QF-based algorithm successfully matches cell subsets that are significantly shifted between samples. Panel a: to identify basophils, we used the following gating sequence^10^: FSС-A/SSC-A (total white blood cells) → FSС-A/FSC-H (singlets) → CD41a/live/dead (CD41a–live) → Dump [CD3, CD66b, HLA-DR]/CD123 (Dump–, CD123++)]. The dataset for these 3 patients is available at https://flowrepository.org/id/FR-FCM-ZY3B Panel b: to identify CD34+ CD45mid cells, we reproduced the gating strategy presented on Data S3B in^14^ using DBM clustering algorithm^12^. H4 is a healthy control sample, SJ1 and SJ16 are AML patients’ samples. Mass cytometry data corresponding to this example are publicly available at https://www.cytobank.org/nolanlab/reports/Levine2015.html (CyTOF AML PhenoGraph manually gated CD34 x CD45 AML blast populations, Data S2E).

In the first example, we used fluorescence flow cytometry dataset collected in the frames of basophils activation study^13^. Basophil marker expression levels commonly differ from sample to sample. In the original study^13^, the authors use the surface level of CD123 as a phenotypic marker to identify peripheral blood basophils. The expression of this marker commonly varies from patient to patient (e.g., in Fig. 5a, MFI varies from 1033 to 6672). Importantly, QFMatch is capable of aligning such clusters, even when MFI values and the size of the basophil vary from one sample to another. Basophil populations in these patient samples have relative frequencies of the same order of magnitude (Fig. 5a) while the distance between their geometric means is significant ([0.6; 0.7]σ when comparing Patient 1 with Patient 2 and [5.1; 1.6]σ between Patient 1 and Patient 3).

In the second example, we used a mass cytometry dataset collected in an acute myeloid leukemia (AML) pathophysiology study^14^. In the original study, the authors quantitated CD34+Cd45mid cells in five healthy controls (H) and sixteen AML patients (SJ). Both the relative frequiency of CD34+ Cd45mid cells and the expression of these markers significantly vary between healthy controls and patients, and from patient to patient (in the representative example shown on Fig. 5b, CD34 MFI varies from 36 to 144; CD45 MFI varies from 74 to 500; relative frequency of CD34+Cd45mid cells varies from 4.7% to 58%). QFMatch is capable of aligning such clusters, even when MFI values and the frequencies of the CD34+Cd45mid cells vary from one sample to another.

### Detection of missing lymphocyte populations in the peritoneal cavity of *RAG* knockout (RAG^−/−^) mice

**Figure 6:**
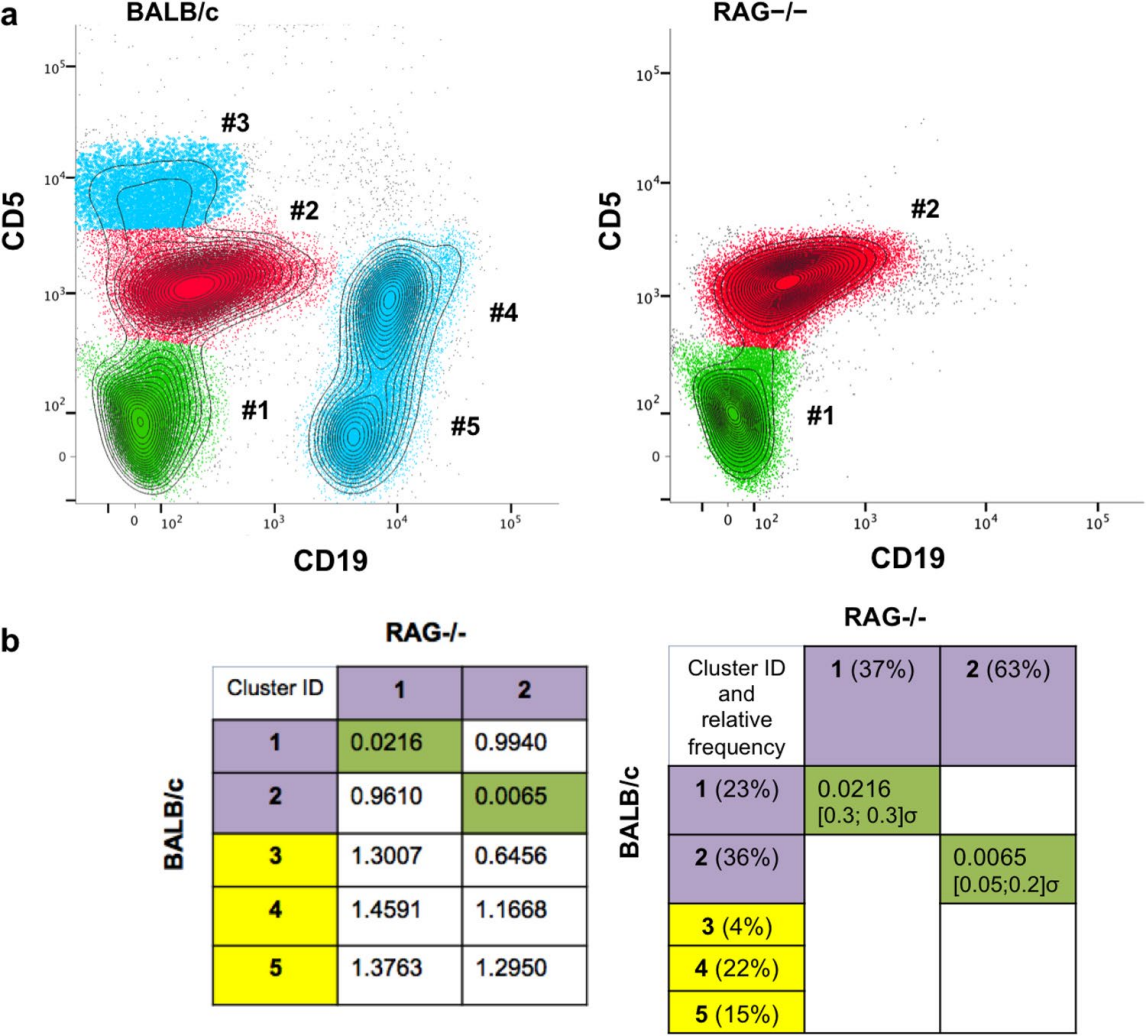
QF dissimilarity scores reveal lack of lymphocyte compartment in RAG^−/−^ mouse. Cells were obtained from the peritoneal cavity of BALB/c (wild-type) and RAG^−/−^ (knockout) mice, stained for surface markers and analysed by flow cytometry (for experiment details, see ref.^18^). We used the following gating strategy (according to^18^): Propidium Iodide^−^(live cells)/FSC-A → FSC-W/FSC-A → CD19/CD5. The clusters highlighted in the same color represent the cell subsets that were matched between BALB/c and RAG^−/−^. Unmatched cell subsets are highlighted in blue (panel a) and their corresponding cluster ids are highlighted in yellow in the table (panel b). This dataset is available at https://flowrepository.org/id/FR-FCM-ZZJF.

We aligned samples of wild-type (BALB/c) and knockout (RAG^−/−^) mouse peritoneal cavity cells (PerC) based on cell surface expression of CD5 and CD19, which respectively indentify T and B lymphocytes, i.e., CD5^hi^CD19^−^ and CD19^hi^CD5^lo^/^−^ (Fig. 6a). We computed QF scores (Fig. 6b) that compare data for the wild-type reference sample (BALB/c) and for the sample that completely lacks T and B lymphocytes (RAG^−/−^). The unmatched clusters were automatically considered as merging candidates by the algorithm. However, this process didn’t decrease any of the initial dissimilarity scores, thus confirm that RAG^−/−^ mice completely lack T and B lymphocytes.

### Cluster matching of the murine lymphoid, myeloid and granuloid lineages between PerC and spleen

**Figure 7:**
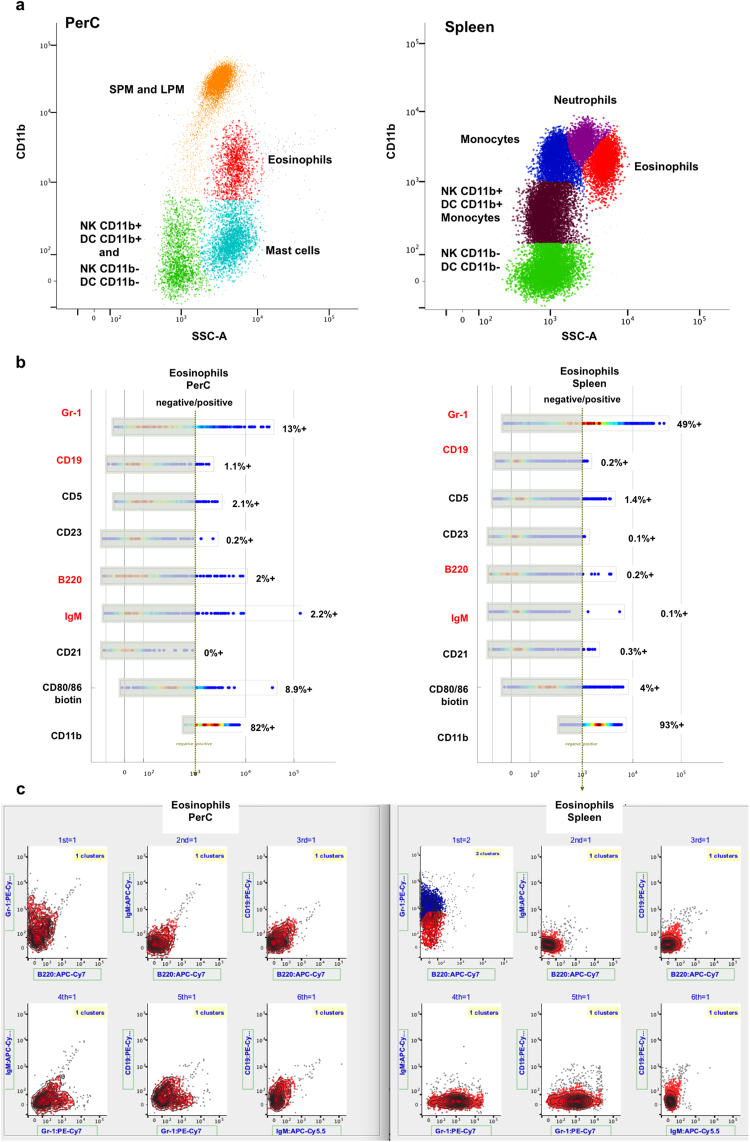
Matching of cell populations between PerC and spleen samples. PerC and spleen from wild-type mouse were processed into a single cell suspension and stained with fluorochrome-conjugated monoclonal antibodies in a 12-parameter Hi-D flow cytometry panel (10-color + Side and Forward Scatter). Data were collected with a Stanford Shared FACS Facility instrument (BD LSRII). Data were then preprocessed, clustered and aligned between samples using AutoGate. The Hi-D panel used in this study identifies the following murine immune cell subsets: lymphoid (NK cells), myeloid (monocytes, macrophages, and dendritic cells), and granuloid (neutrophils, eosinophils and mast cells). Panel a: the clusters highlighted in the same color represent the cell subsets that were matched between PerC and spleen. Unmatched cell subsets are highlighted in blue for the PerC sample and in violet for the spleen sample. We used the following gating strategy (according to^18^): FSC-H/FSC-A (to exclude doublets and clumps) → Propidium Iodide–(live cells)/FSC-A → CD19–/CD5– → SSC-A/CD11b. This dataset is available at https://flowrepository.org/id/FR-FCM-ZY3L. Panel b: we further compared eosinophils populations from PerC and Spleen to determine their global (dis)similarity. First, we used a “Pathfinder” tool provided by AutoGate (http://cytogenie.org/path-finder) to show the staining on all parameters for selected cells. Pathfinder depicts each parameter with a horizontal bar that uses pseudocolor convention to show where the staining is most intense. The vertical dashed line indicates a user definable threshold for positive stain (e.g, based on FMO staining). Pathfinder allows users to quickly scan all of the marker dimensions and choose the most informative (marked in red). In addition, we sequentially matched the eosinophils populations projected in each possible combination of the most informative dimension pairs (panel c, the matched subsets in PerC and Spleen are shown in the same color). This comparison reveals that splenic eosinophils express higher levels of Gr-1 than PerC eosinophils. This data is consistent with the knowledge that expression levels of surface Gr-1 vary by tissue and “inflammatory” condition.

Using a dataset for side scatter (SSc-A, which correlates with cell granularity) and CD11b surface marker measures, we matched mouse PerC and spleen samples to explore differences in the representation of the lymphoid, myeloid and granuloid subsets. This is a good case to test the cluster matching algorithm because the type of immune cells present in PerC and spleen are quite different from one another, i.e., naïve spleen lacks virtually all mast cells and small and large peritoneal macrophages (SPM and LPM, respectively) whereas naïve PerC have very few monocytes and neutrophils but still share some types of immune cells, including dendritic cells (DC), natural killer cells (NK), and eosinophils. Note that the QFMatch algorithm successfully aligned the immune cell subsets that are shared between spleen and PerC (marked in the same colors in Fig. 7) and detected the missing cell subsets (marked in different colors in Fig. 7).

## Discussion

Population matching is one of the most important analytical tools used in the flow/mass cytometry data analysis pipeline in a variety of research/clinical settings. Recently developed cluster matching methods intended for this purpose can be informally divided into two types:

1. *Separate clustering and matching*. This type of approach, used for example in FLAME^4^ and PhenoGraph^14^, identifies cluster locations in each individual sample (e.g., by using mixture models or by constructing a graph and using modularity optimization to cluster it). It then pools these cluster locations for all of the samples in a given class, for example “healthy controls”, and clusters again (e.g., by using partitioning around medoids or by constructing a graph from pooled pre-clustered data) to construct a Hi-D template of meta-clusters.
  As discussed in detail in^15^ and^7^, this type of approach is likely to fail when the population pattern varies significantly between samples (i.e., population locations differ significantly or even (dis)appear from sample to sample). Thus, a meta-cluster corresponding to distinct cell subsets can split into multiple sub-clusters if extraneous clusters appear in one (or more) of the samples. Further, graph-based methods, such as PhenoGraph^14^ can fail to assign phenotypically distinct cell subsets to distinct meta-clusters (Fig. 8b). Finally, because the partitioning of individual samples into clusters is performed independently from sample to sample, even quite similar samples may be poorly aligned.

**Figure 8:**
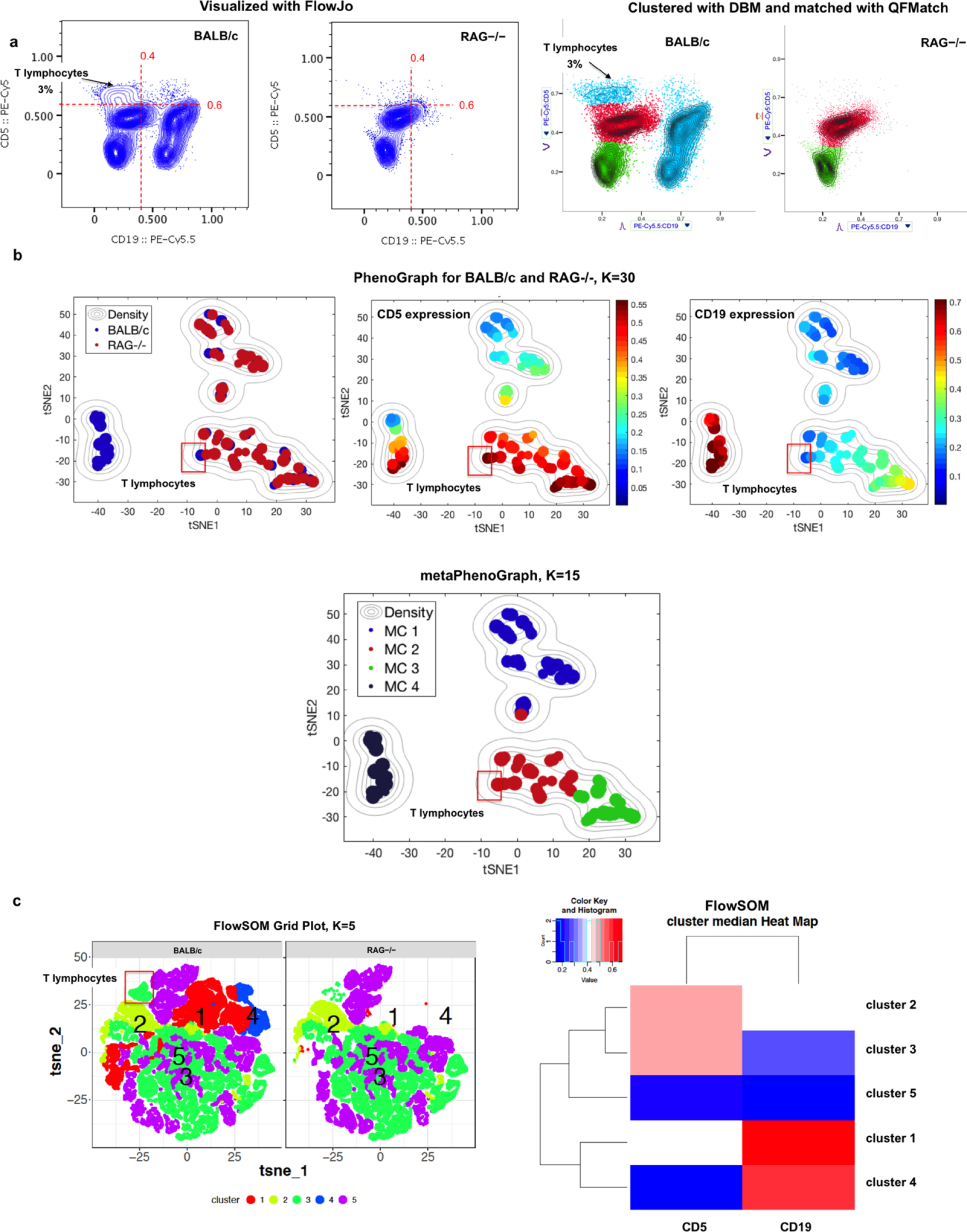
PhenoGraph and FlowSOM meta-clustering approaches fail to reveal the absence of T lymphocytes in RAG−/− mice. Panel a shows downsampled datasets (30 k for each) obtained from the samples shown in Fig. 6 (data are Logicle-transformed and visualized on linear scales). The right side of panel a shows results for QFMatch algorithm. These data were used (panels b and c) to determine whether PhenoGraph^14^ and FlowSOM^16^ reveal distinct meta-clusters (MCs) corresponding to T lymphocytes (CD5 high and CD19 negative) in BALB/c peritoneal cells. The clustering and meta-clustering results are shown for the default values of the input parameters (PhenoGraph: K = 30 for clustering step and K = 15 for meta-clustering step; FlowSOM: K = 5). We also tested (clustering results are available here: https://www.dropbox.com/sh/wehjkb223jlgf04/AAD9D3Ujx_r0r3H5dR9Uuj5Ia?dl = 0 and https://drive.google.com/drive/folders/1-Tm0eyOH4ZsN9fLbMr6_wvy6iQ4XgT0p) the ability of PhenoGraph and FlowSOM to assign T lymphocytes subset to a distinct meta-cluster using different combinations of the input parameters (PhenoGraph: K = 5; 15; 30; 45; 100 for clustering and K = 3; 5; 15; 30 for meta-clustering; FlowSOM: K = 5; 10; 15). None of these parameters combinations resulted in correct MCs identification. For these studies, we used PhenoGraph code and default settings provided by Dana Pe’er and colleagues^21^. To run FlowSOM, we used the Cytofkit core function included in the Bioconductor package^22^. With these implementations, it took PhenoGraph about 1.5 minutes to cluster and align clusters between BALB/c and RAG−/− mice samples in 2 dimensions, and it took FlowSOM about 10 minutes, both on a computer running Mac OS X 10.11.6, with 3.1 GHz Intel Core i7 and 16 GB of RAM. The data shown were initially compensated and transformed with Logicle utilities provided by AutoGate (http://CytoGenie.org/).
2. *Joint clustering and matching*. This type of approach, including Joint Clustering and Matching^6,15^, FlowSOM^16^ and ASPIRE^7^, aligns cell populations based on direct modelling of contributions from individual and grouped samples. It can be thus considered as “hierarchical extension of statistical mixture models”, since it applies a hierarchical (multi-level) model that incorporates information from both the individual and group levels when fitted to flow cytometry data. In these settings, an individual sample is considered a noisy realization of a more general biological population mixture. For example, individual samples could be modeled using a Dirichlet process Gaussian mixture model approach^17^ and linked through hierarchical prior. As we show on Fig. 8c, joint clustering and matching approaches, such as FlowSOM^16^, can fail to assign phenotypically distinct cell subsets to distinct meta-clusters.

Both of these types of cluster matching methods rely heavily on fitting mathematical models to identify and match clusters. Thus, they are dramatically hindered by the curse of dimensionality because the number of combinations of parameters increases dramatically as the number of dimensions increases above three or four. Additionally, these methods are quite computationally demanding and often rely on a heuristic to tune a set of input parameters (see Fig. 8).

To address the key problems mentioned above, we improve on a principle shared by most existing cluster matching methods, i.e., the use of (dis)similarity measures between cell populations. Most current methods rely on different types of (dis)similarity measures, including (1) Joint Clustering and Matching^6^, which is based on a symmetric form of the Kullback-Leibler (KL) divergence; (2) a flowMatch package^5^, which employs Euclidean distance, Mahalanobis distance and KL divergence for computing the dissimilarities between clusters; and, (3) another commonly used package, FLAME^4^, which relies on a solution of minimum cost bipartite matching (essentially minimum Euclidean distance and corresponding weights difference to solve).

In our previous paper^10^, we argued that in order to be biologically/biomedically informative, the (dis)similarity measure should satisfy the following criteria: (1) it must possess the properties of a metric (non-negative symmetric functions that satisfy the triangle inequality and the axiom of coincidence); (2) it should distinguish biologically significant differences from small differences due to instrument drift or other irrelevant factors; (3) it should be non-parametric, to account for the complex structure of the cell populations commonly found in flow cytometry data; and, (4) it should be computationally efficient, so that modern high throughput analyses can be performed quickly. However, constraint #2 (the need to distinguish biologically significant differences) is the most critical for flow cytometry and similar datasets. This constraint basically rules out most of the current approaches.

In^10^, we demonstrated that distance metrics (e.g., Earth Mover’s Distance (EMD)), which take into account changes in both location and frequency rather than just changes in one or the other, are the most suitable and accurate methods for comparing multivariate non-parametric flow cytometry data distributions. However, EMD is computationally complex. Further, the algorithm can be slow for practical applications of cluster matching tasks in Hi-D flow cytometry.

To overcome this speed limitation, we developed the computationally efficient QF-based method (QFMatch) defined here, which takes changes in location and frequency into account and is insensitive to small changes caused by instrument noise. QFMatch also satisfies criteria (1)-(4) discussed above.

Applying the QF distance measure to flow cytometry data was originally suggested by Rajwa’s group^3^. However, this group developed the method only for a one-dimensional case and didn’t apply it directly to cluster matching tasks. Here, we have further developed the method and made it applicable to Hi-D flow and mass cytometry cluster matching tasks.

The QF approach described here can be used with any number of dimensions since it is based on the adaptive binning that avoids the curse of dimensionality by recursively splitting sample along the axis with the highest variation. Thus, the dimensionality enters in the computation at most linearly. Furthermore, computing the QF does not depend on the dimensionality at all, as in the QF-based algorithm we just sum over bins. Therefore, computing the QF does not suffer from the computational curse of dimensionality.

The QF approach can be used with any method that enables valid identification and isolation of cellular (or other) subset in which markers are expressed. Typically, this clustering task is subject to the curse of dimensionality. We avoid this curse here by coupling the QF-based cluster matching method with a two-dimensional density-based merging (DBM) clustering algorithm^12^.

We have now implemented QFMatch in a provisional flow cytometry data analysis package that we make freely available (no charge) at CytoGenie.org to users at non-profit organizations (e.g.,.edu and.gov). In its current implementation, QFMatch aligns cell clusters across a pair of samples. However, this method can be further extended to work with large collections of samples, e.g., the QF dissimilarity measure can be used to construct templates of meta-clusters for samples that belong to one class and can further be used to align these templates of meta-clusters between classes.

## Materials and Methods

### Experiment overview

We use QF to match subsets between relevant samples (same staining panels) within the biological/biomedical datasets described below.

### Flow sample description

Human and mouse datasets shown in Figs 5 and 6 were generated in previously published studies (see refs^13,14,18^ for complete materials and methods). Access to the data was provided by the investigators responsible for the studies. Human subject guidelines are described in Gernez *et al*.^13^ and Levine *et al*.^14^. Patient records/information was anonymized and de-identified prior to acquisition for these studies.

Mouse PerC and spleen datasets shown in Fig. 7 were explicitly generated for this study using adult (>8 wks) naïve wild-type C57BL/6 strain. Mouse studies were approved by the Stanford Animal Care and Use Committee and are in compliance with the Administrative Panel on Laboratory Animal Care guidelines. Peritoneal cells were harvested by injecting 6 ml of custom RPMI-1640 media into the peritoneal cavity. Spleens were mechanically disrupted to obtain single cell suspension. Cells were filtered over a 70 µm nylon filter and erythrocytes were lysed using ACK buffer. Cells were resuspended at 100 × 10^6^ cells/ml and stained on ice for 30 min with a reagent panel that detects a total of 12 parameters. Stained cells were resuspended in 10 ug/ml propidium iodide (PI) to enable exclusion of dead cells.

Datasets presented on Figs 5a, 6 and 7 were stored immediately after collection into a stable long-term archive maintained by the Stanford Shared FACS Facility. See figure legends for gating strategy.

### Instrument details

Information about instruments used to collect human and mouse samples can be found in^13,14,18^. PerC and Spleen cells were analyzed on Stanford Shared FACS Facility instruments (BD LSRII) equipped with 4 lasers (405 nm, 488 nm, 532 nm, and 640 nm) and 19 PMTs. Data were collected for 0.2 × 10^6^ to 2 × 10^6^ cells.

### Data analysis details

The proposed workflow for analyzing all four datasets used in this manuscript consists of two steps:

1. Preprocess the data by sequentially using utilities available in AutoGate^19^ (http://CytoGenie.org/) to compensate the data (fluorescence flow cytometry data only), transform it with the Logicle transformation^20^, and cluster the transformed data with DBM^12^. See figure legends for gating sequences. The flow cytometry data prepocessing methods used here do not require user input for parameters such as number of clusters, number of grid bins, manual gating for compensation purposes, etc.
2. Use QF to match cell populations of interest, for example, populations of eosinophils (see Fig. 7). The QF-based cluster matching algorithm is integrated into AutoGate (http://CytoGenie.org/).

Combining Logicle transformation, DBM for cell population identification, probability binning, and QF provides a complete pipeline for cluster matching of flow cytometry samples. However, we would emphasize that the QF approach for cluster matching of flow cytometric subsets works independently of how the population was defined here. For example, the clusters could be defined by using domain knowledge-driven manual gating, a sequential automated clustering approach, or a simultaneous clustering approach.

### Data Availability

The datasets generated during and/or analysed during the current study are available in the FlowRepository and Cytobank:

https://flowrepository.org/id/FR-FCM-ZY3B, https://www.cytobank.org/nolanlab/reports/Levine2015.html (CyTOF AML PhenoGraph manually gated CD34 x CD45 AML blast populations, Data S2E).

https://flowrepository.org/id/FR-FCM-ZZJF

https://flowrepository.org/id/FR-FCM-ZY3L.
